# Histopathological predictor of response to mirikizumab in active ulcerative colitis

**DOI:** 10.1007/s12328-026-02298-0

**Published:** 2026-03-06

**Authors:** Ichitaro Horiuchi, Akira Horiuchi, Kaori Horiuchi

**Affiliations:** 1 Department of Gastroenterology, Shinshu University Hospital, Matsumoto, Japan; 2 Digestive Disease Center, Showa Inan General Hospital, 3230, Akaho, Komagane, 399-4117 Japan

**Keywords:** ulcerative colitis, mirikizumab, epithelial neutrophilic infiltration, IL-23

## Abstract

This case series reports three patients with active ulcerative colitis (UC) successfully treated with mirikizumab, a selective IL-23p19 monoclonal antibody. Mirikizumab was initiated based on both clinical presentation and a histopathological marker—high neutrophilic infiltration of the colonic epithelium classified as Geboes Grade 3.2—which reflects an IL-23/Th17-driven inflammatory phenotype. The cases included: an elderly man with acute severe pancolitis and primary nonresponse to infliximab; a middle-aged woman with steroid-dependent left-sided UC and inadequate response to adalimumab and ustekinumab; and a young biologic-naïve woman with steroid-refractory UC and bowel urgency. All three patients demonstrated high epithelial neutrophilic infiltration (Geboes Grade 3.2). These cases demonstrate the potential utility of histology-guided biologic selection and support Geboes Grade 3.2 as a candidate predictive biomarker for IL-23 inhibitor response in UC.

## Introduction

Therapeutic strategies for moderate-to-severe ulcerative colitis (UC) increasingly integrate clinical history and prior biologic exposure to personalize selection of advanced therapies [1]. Histopathological characterization, particularly the detection of epithelial neutrophilic infiltration using the Komagane evaluation method—a modification of the Geboes scoring system—is emerging as a potentially relevant factor to guide biologic choice [2, 3]. The Komagane method is an institutional modification of the Geboes score, which we previously described and validated in a recent study [2, 3]. The original Geboes Grade 3 was subdivided into four subcategories based on the extent of neutrophilic infiltration: 3.0 (0%), 3.1 (< 5%), 3.2 (5–50%), and 3.3 (> 50%) [4]. The percentage of crypts with neutrophilic infiltration is calculated by dividing the number of infiltrated crypts by the total number of crypts on a glass slide. To reduce interobserver variability, two independent pathologists with different levels of expertise in gastrointestinal pathology performed the assessments. This approach improves the reliability and reproducibility of the quantitative evaluation of neutrophilic infiltration compared with the original Geboes scoring system [4]. Agents that target the IL-23 pathway, such as mirikizumab, a selective IL-23p19 monoclonal antibody, may be particularly effective in inflammatory contexts that are Th17-predominant and characterized by high neutrophilic infiltration [5]. These agents may benefit patients who have an inadequate response to anti-TNF agents or ustekinumab, as well as select biologic-naïve individuals with active inflammation. This case series indicates that high epithelial neutrophilic infiltration (Geboes Grade 3.2) may function as a biomarker for an IL-23/Th17-predominant phenotype and predict a favorable response to the selective IL-23p19 inhibitor, mirikizumab.

## Case reports

Case 1: Acute severe pancolitis with primary nonresponse to infliximab

**Fig. 1:**
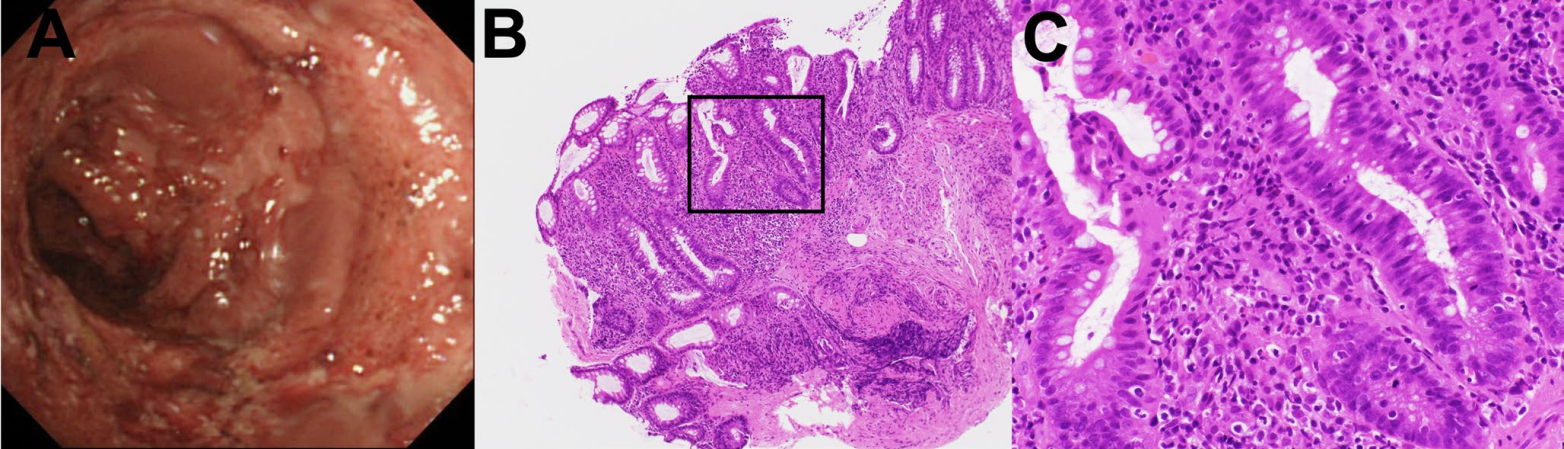
Pre-treatment findings in Case 1. **a** Colonoscopy revealing severe inflammation with deep ulcerations (Mayo Endoscopic Subscore 3). **b** Low-magnification histopathology demonstrating marked inflammatory infiltrate (hematoxylin and eosin stain). The black frame indicates the high-magnification area. **c** High-magnification view demonstrating high neutrophilic infiltration of the crypt epithelium (Geboes Grade 3.2)

A 79-year-old man with extensive pancolitis presented with a severe relapse (Clinical Activity Index [CAI] 15) despite standard induction therapy with infliximab (5 mg/kg, three doses), with no clinical improvement. Steroid therapy was not used prior to the induction of infliximab due to the patient’s advanced age, as there was concern for adverse events. Colonoscopy revealed Mayo Endoscopic Subscore 3. Histopathological examination of colonic biopsies from inflamed areas demonstrated Geboes Grade 3.2, characterized by high neutrophilic infiltration of the crypt epithelium (Fig. 1). At the time of infliximab induction, the potential for histological evaluation to guide future mirikizumab therapy was not yet established. Based on this histopathological pattern and primary nonresponse to infliximab, mirikizumab was initiated with an induction regimen of 300 mg intravenously every four weeks for 12 weeks, followed by subcutaneous maintenance therapy at 200 mg every four weeks. The patient achieved clinical remission within four weeks and endoscopic remission after six months. He has been in endoscopic remission for two years with mirikizumab treatment alone.

Case 2: Steroid-dependent left-sided colitis with secondary loss of response to biologics.

**Fig. 2:**
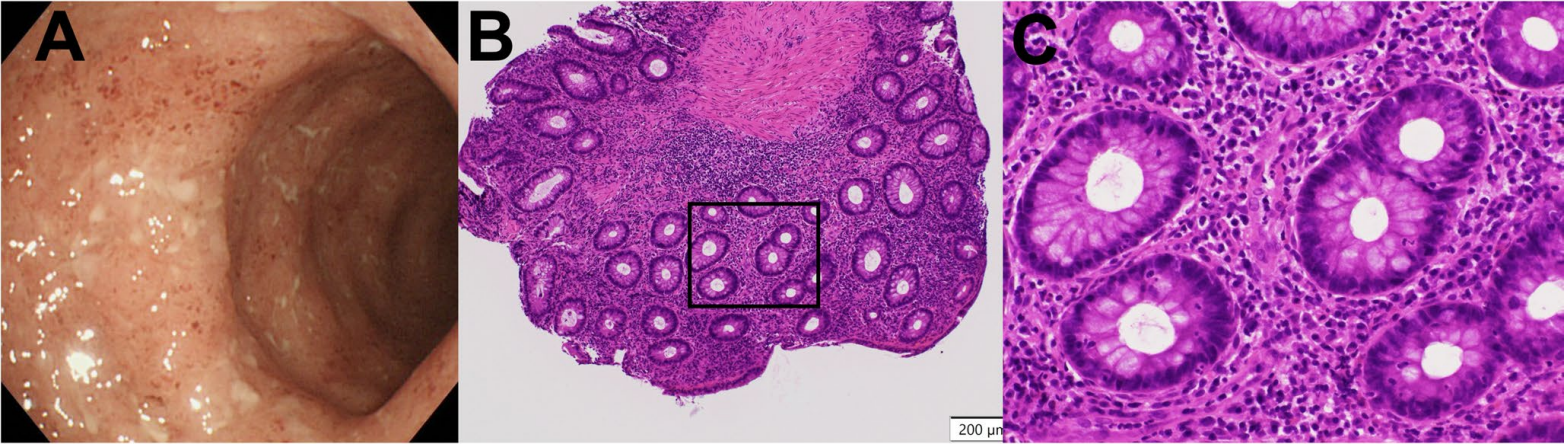
Pre-treatment findings in Case 2. **a** Colonoscopy revealing moderate inflammation with absent vascular pattern, friability and diffuse erosions (Mayo Endoscopic Subscore 2). **b** Low-magnification histopathology demonstrating moderate inflammatory infiltrate (hematoxylin and eosin stain). The black frame indicates the high-magnification area. **c** High-magnification view demonstrating high neutrophilic infiltration of the crypt epithelium (Geboes Grade 3.2)

A 44-year-old woman with a three-year history of left-sided UC remained persistently steroid-dependent. She experienced disease relapse after six months of adalimumab therapy. Subsequently, after one year of ustekinumab (every eight weeks), she developed worsening disease activity, with her CAI increasing from 3 to 8. Colonoscopy revealed Mayo Endoscopic Subscore 2. Biopsies evaluated using the Komagane method demonstrated Geboes Grade 3.2 with high epithelial neutrophilic infiltration (Fig. 2). The importance of histological evaluation during ustekinumab maintenance therapy in determining an appropriate mirikizumab induction regimen has not been recognized. This histopathological pattern, combined with inadequate response to adalimumab and ustekinumab and ongoing steroid dependence, supported transition to mirikizumab using the induction and maintenance regimen described in Case 1. Conventional induction therapies, including tacrolimus and granulocytapheresis were not selected when mirikizumab was initiated. She achieved clinical remission, defined by resolution of rectal bleeding and normalization of stool frequency, within four weeks and endoscopic remission at six months. She has been in clinical remission for two years by using mirikizumab alone.

Case 3: Biologic-naïve steroid-refractory left-sided colitis

**Fig. 3:**
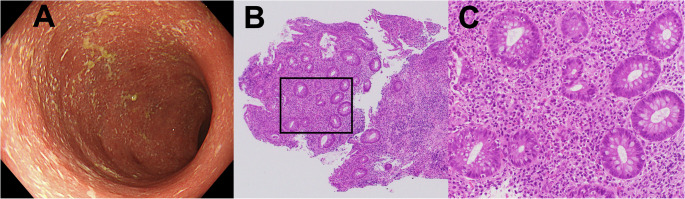
Pre-treatment findings in Case 3. **a** Colonoscopy revealing moderate inflammation with loss of vascular pattern, erythema and diffuse erosions (Mayo Endoscopic Subscore 2). **b** Low-magnification histopathology demonstrating marked inflammatory infiltrate (hematoxylin and eosin stain). The black frame indicates the high-magnification area. **c** High-magnification view demonstrating high neutrophilic infiltration of the crypt epithelium (Geboes Grade 3.2)

A 30-year-old biologic-naïve woman with a one-year history of left-sided UC demonstrated inadequate response to 5-aminosalicylic acid maintenance therapy and oral prednisolone 30 mg/day. She presented with severe diarrhea and bowel urgency (CAI 9). Colonoscopy revealed Mayo Endoscopic Subscore 2. Biopsies from the most inflamed regions demonstrated Geboes Grade 3.2 with high neutrophilic infiltration of the crypt epithelium (Fig. 3). Given her severe steroid-refractory disease and biologic-naïve status, mirikizumab was selected as first-line biologic therapy based on the histopathological findings. Alternative induction therapies, such as other biologics, tacrolimus, or granulocytapheresis, were not chosen. She achieved clinical remission within four weeks and endoscopic remission at six months. She has also remained in clinical remission for two years by using mirikizumab alone.

## Discussion

All three patients demonstrated high epithelial neutrophilic infiltration (Geboes Grade 3.2), corresponding to Th17-predominant mucosal inflammation. This inflammatory pattern is mechanistically driven by the IL-23/Th17 axis and downstream effector cytokines such as IL-17 A and IL-17 F [6–8]. High epithelial neutrophilic infiltration may therefore serve as a surrogate marker for IL-23-mediated inflammatory processes and support the selection of mirikizumab [2, 3, 9]. Our clinical approach is based on the following principle: when any biopsy from an active site demonstrates Geboes Grade ≥ 3.2, whether at initial diagnosis or disease relapse, IL-23 inhibitor therapy may be considered. This approach proved successful in our cases with anti-TNF primary nonresponse, secondary loss of response to ustekinumab, and biologic-naïve steroid-refractory disease. This strategy aligns with long-term efficacy data from the LUCENT-3 extension study demonstrating sustained mirikizumab effectiveness [10].

Notably, Case 2, who experienced secondary loss of response to ustekinumab (an IL-12/23p40 inhibitor), subsequently responded to mirikizumab (a selective IL-23p19 inhibitor). Persistent neutrophilic crypt injury despite p40 inhibition may indicate that more selective p19 blockade is required, potentially offering enhanced disruption of Th17-mediated inflammation. In addition, follow-up biopsy samples taken from the same segments during endoscopic remission revealed a marked decrease in neutrophilic infiltration, improving to Geboes Grade 3.0 or 3.1 in all three cases. Our cases were classified as Grade 3.2, but we believe that Grade 3.3, which shows even more extensive crypt destruction and neutrophilic load, would also benefit from IL-23p19 blockade because it likely reflects severe IL-23-driven pathology.

The main limitations of this case series include the small sample size and absence of a control group. These findings should therefore be regarded as hypothesis-generating and are insufficient to establish causality. We also acknowledge a limitation of the Komagane method; specifically, the requirement for time-consuming quantitative crypt counting by pathologists may restrict its utility in routine clinical settings. In 2023, risankizumab and guselkumab, which are other IL-23p19 inhibitors that act similarly, were unavailable in Japan.

In summary, Geboes Grade 3.2 neutrophilic infiltration may represent a candidate predictive biomarker for response to IL-23p19 inhibition with mirikizumab in patients with active UC, including those who are biologic-naïve or have demonstrated inadequate response to anti-TNF agents or ustekinumab. Although preliminary data indicate that severe neutrophilic infiltration (Geboes Grade 3.2) correlates with a favorable response to mirikizumab, the limited sample size necessitates caution. Therefore, histological assessment should be considered an adjunctive tool rather than a definitive determinant for initiating therapy at this stage. The proposed histology-guided therapeutic strategy requires validation in larger prospective controlled trials.
